# HLA-B and cysteinylated ligands distinguish the antigen presentation landscape of extracellular vesicles

**DOI:** 10.1038/s42003-021-02364-y

**Published:** 2021-07-01

**Authors:** Julia Bauzá-Martinez, Albert J. R. Heck, Wei Wu

**Affiliations:** 1 Biomolecular Mass Spectrometry and Proteomics, Bijvoet Center for Biomolecular Research and Utrecht Institute for Pharmaceutical Sciences, Utrecht University, Utrecht, The Netherlands; 2 Netherlands Proteomics Centre, Utrecht, The Netherlands

**Keywords:** Peptide vaccines, Antigen processing and presentation

## Abstract

Extracellular vesicles can modulate diverse processes ranging from proliferation and tissue repair, to chemo-resistance and cellular differentiation. With the advent of tissue and immunological targeting, extracellular vesicles are also increasingly viewed as promising vectors to deliver peptide-based cancer antigens to the human immune system. Despite the clinical relevance and therapeutic potential of such ‘cell-free’ approaches, the natural antigen presentation landscape exported in extracellular vesicles is still largely uncharted, due to the challenging nature of such preparations and analyses. In the context of therapeutic vesicle production, a critical evaluation of the similarity in vesicular antigen presentation is also urgently needed. In this work, we compared the HLA-I peptide ligandomes of extracellular vesicles against that of whole-cells of the same cell line. We found that extracellular vesicles not only over-represent HLA-B complexes and peptide ligands, but also cysteinylated peptides that may modulate immune responses. Collectively, these findings describe the pre-existing provision of vesicular HLA complexes that may be utilized to carry peptide vaccines, as well as the propensity for different peptide and post-translationally modified ligands to be presented, and will outline critical considerations in devising novel EV vaccination strategies.

## Abstract (web summary)

Bauzá-Martinez et al. analyzed the HLA-I peptide ligandome in extracellular vesicles (EVs) from an EBV-immortalized B cell line, JY, and compare HLA-I-binding peptides recovered from EVs to those recovered from whole-cells. The authors report an enrichment for HLA-B-binding peptides and for post-translationally modified cysteinylated peptides in EVs as compared to whole-cells.

## Introduction

Cancer immunotherapy has advanced substantially in the last decade, not only with breakthroughs involving checkpoint blockade inhibitors^1^, but also due to conceptual advances in antitumor vaccines. To date, vaccinology approaches directed at eliciting antitumor effects using the patient’s own immune system have shown great promise in a variety of cancers such as melanoma^2–4^, non-small cell lung carcinoma^5,6^, and glioblastoma^7^.

In practice, antigenic vaccines need to be loaded on antigen-presenting cells (APCs) such as dendritic cells. This can be done exogenously before injection of these loaded APCs into the patient, or antigens may also be infused into the patient blood as carried by extracellular vesicles (EVs), to take advantage of the natural EV uptake^8^ by APCs that remain in the body^9^. The latter is often referred to as “cell-free vaccines”^10,11^, which has been shown to be more immunogenic than introducing soluble antigens^12^, likely due to the trained uptake of EVs by APCs. Via either approach, the ultimate goal is for these antigenic signals to reach the lymph node, the primary site of T cell priming, so as to kickstart activation and expansion of clonal T cell populations, which may, in turn, be reactive against the patient’s own tumor^11,13–17^.

Apart from RNA-based therapeutics delivered via EVs^18–21^, which are already promising, peptide-based antigens may mimic the normal antigenic processes in the body more closely, as if a real tumor-specific peptide is presented. The superior stability of peptide vaccines is also desirable when considering the shelf-life and formulation of such anti-cancer therapeutics. From screening the EV contents of a large panel of cell lines^22^, the conserved presence of HLA proteins in EVs was also noted. Collectively, these allude to an exciting possibility to deliver antigens by taking advantage of these EV HLAs.

With recent advances in mass spectrometry-based immunopeptidomics that we and others have contributed^23–25^, greater depth has been achieved in HLA peptide profiling from cell lines, tissue specimen^26–29^ and increasingly scarce and exotic material^30^. Nevertheless, to our knowledge, only one publication has so far reported the identification of ~500 HLA peptide ligands eluted from EVs^31^. This limited exploration into the EV HLA peptide ligandome stems from the challenges of obtaining sufficient EV material, on top of the need for extensive purifications before immunoaffinity capture of HLA complexes. Yet, gaining more knowledge into the EV HLA peptide repertoire is critical to tap on the possibility of tailored antigen delivery. In this respect, specific questions arise, for instance, the resemblance between EV and whole-cell HLA peptide ligandomes in terms of repertoire and post-translational modifications, as well as whether there are specific characteristics of peptides that are prone to become presented via the EVs. Outlining such rules will guide the intelligent design of novel EV vaccination strategies.

Using a highly sensitive workflow adapted from our established HLA-I peptide purification method^24^, we compared the EV HLA-I peptide ligandome against the whole-cell HLA-I peptide ligandome of the same cells. Such a direct comparison is much needed to establish unique features of EV HLA peptide presentation, that could be therapeutically exploitable. Our study described here led to the identification of ~3500 HLA-I peptide ligands, many of which were uniquely detected from EVs. We also observed that HLA-B peptide binders and peptides containing cysteinylated cysteines were more prominently presented by the EV HLA complexes. Taken together, these findings describe the preexisting provision of HLA complexes on EVs and the propensity for different peptide ligands and modified ligands to be presented on EV HLA-I complexes. Such data outlines critical considerations in devising novel EV vaccination strategies and would be a valuable resource for the tumor immunology community.

## Results

### Isolation and characterization of JY-derived extracellular vesicles

**Fig. 1:**
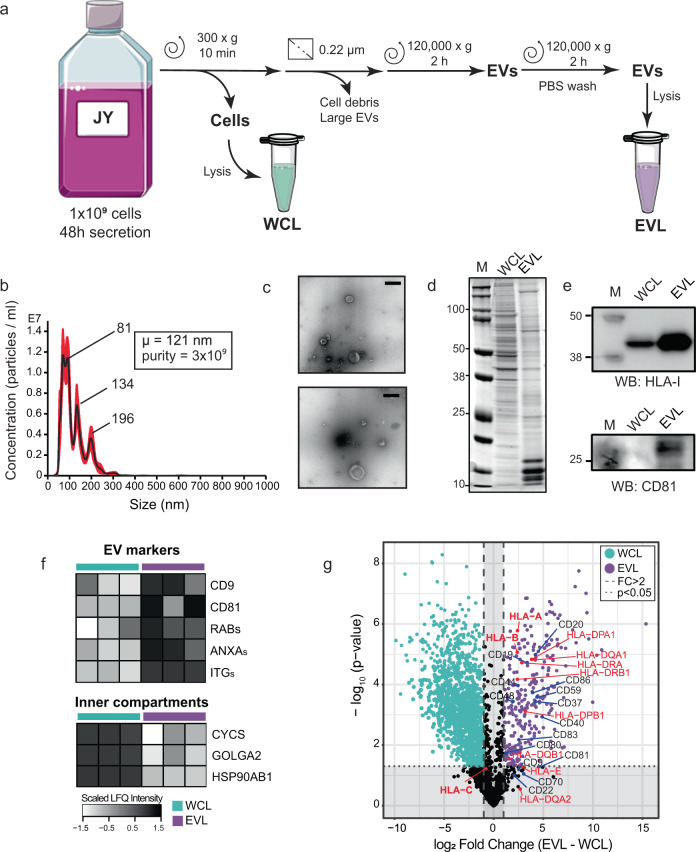
Extracellular vesicle isolation and characterization. **a** Schematic workflow of extracellular vesicle (EV) isolation. Lysates made from JY WC (WCL, green) and JY-derived EVs (EVL, purple) were used subsequently as input for immunoaffinity purification of HLA complexes (Figs. 2–4). **b** Nanoparticle tracking analysis (NTA). Size and concentration of isolated EVs were estimated. Purity (particle:protein ratio) of EVs was 3e9, with mean particle size of 121 nm. Representative trace. **c** Negative stain transmission electron microscopy (NS-TEM) analysis. Morphology of the isolated EVs were cup-shaped. Representative images. Scale bar: 200 nm. Full images provided in Supplementary Fig. 6. **d** SDS-PAGE of protein band patterns in WCL and EVL. Dominant protein bands in WCL and EVL were mutually exclusive. Full image provided in Supplementary Fig. 7. **e** HLA-I and cd81 western blot. From 5 µg load of EVL and WCL, prominent enrichment of HLA-I and CD81 were observed in EVL. Full images provided in Supplementary Fig. 8. **f** Quantitative proteomics analysis of WCL (*n* = 3) and EVL (*n* = 3). Numerous classical EV markers were enriched in EVL. **g** Quantitative proteomics comparison of EVL and WCL. HLA proteins, numerous cell surface CD proteins and tetraspanins were significantly enriched in EVL. Dashed lines indicate proteins with a 2-fold change in abundance, while dotted lines indicate proteins with *p* value ≤ 0.05.

To compare the HLA-I content and contrast the HLA Class I peptide repertoire in EVs and whole-cells (WC), we first prepared and characterized JY WC and EV material (Fig. 1a). In order to preserve the biological validity of direct comparisons between these two sources of HLA-I proteins and HLA class I peptide ligands, JY WC and JY secreted EVs from the same culture batch were always used in each paired analysis. As described in the “Materials and Methods”, EV-depleted culture media was replaced after 24 h to harvest a second batch of EVs, to ensure the cultured cells remain well-supplied with nutrients and were not apoptotic. Both EV harvests were then pooled for subsequent use to eliminate variabilities due to cell density. From a total of 800 ml JY conditioned media, at least 150 µg of EV proteins were obtained for further characterization by nanoparticle tracking analysis (NTA), negative stain transmission electron microscopy (NS-TEM), SDS-PAGE, western blotting, and label-free quantitative proteomics, before this material was used as starting material for HLA-I protein and HLA class I peptide ligand retrieval (Fig. 1b–g).

The EV isolate was high in both purity and integrity, as demonstrated by a range of biophysical and proteomics approaches. Through NTA (Fig. 1b), the JY EVs we isolated were on average about 120 nm in diameter. This is well within the expected range of 50–150 nm range to be classified as exosomes. The EV preparation was almost devoid of particles exceeding 300 nm in size, verifying that the isolates were pure and free from cell debris contaminants and apoptotic bodies. The EVs were also cup-shaped in NS-TEM (Fig. 1c), in strong agreement with documented classical morphology that is characteristic of dehydrated EVs^32^. On lysates of JY WC and EVs, we also verified that dominant proteins in the EVs were distinct from dominant proteins in JY WC (Fig. 1d), and that CD81, a classical EV marker, was strongly enriched in EV lysates and below detection in the whole-cell lysate (Fig. 1e). Together these data demonstrate that EVs were specifically isolated, with no visible contaminations of abundant cytoplasmic proteins. A deeper unbiased proteomic comparison of both lysates showed many other classical EV markers were highly enriched in the EV proteome (e.g., Rab GTPases, integrins or annexins), while intracellular compartment markers (e.g., cytochrome c, GOLGA2, HSP90) were depleted in the vesicles (Fig. 1f, g). Collectively, these data provide strong and diverse evidence to show that our EV preparation from JY cells was of high quality, and that these paired JY whole-cell and EV lysates are good and valid starting material to probe differences in the cellular and extra-vesicular HLA class I peptide ligandome.

### EVs and WC derived HLA-I ligands display similar sequence traits

**Fig. 2:**
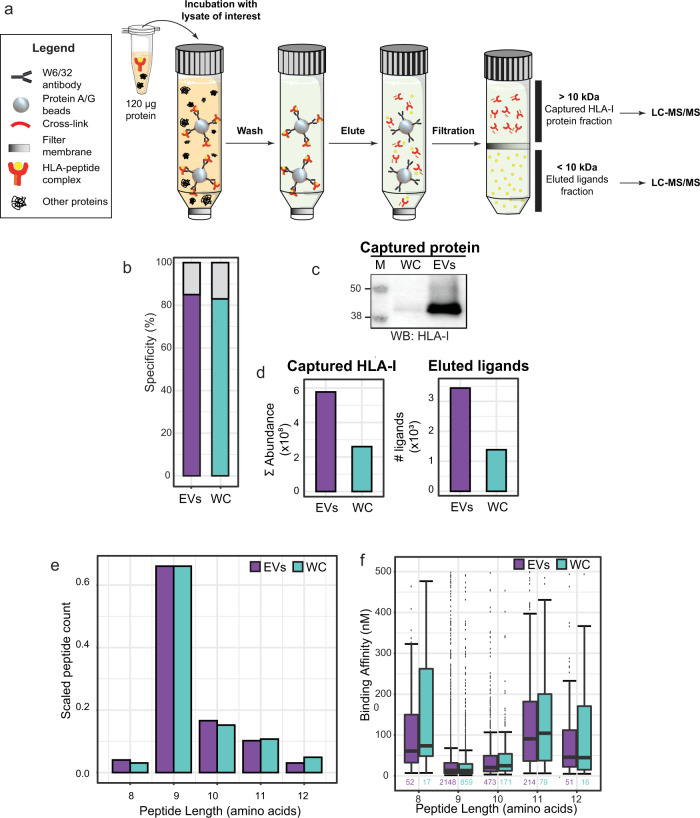
Global characterization of the EV HLA-I peptide ligandome. **a** Preparative steps in the down-scaled HLA-I complex retrieval workflow. An input load of 120 μg was used for immunoaffinity purification of HLA-I complexes from both WCL and EVL. The flowthrough from 10 kDa filtration contains HLA peptide ligands that were subjected to LC-MS/MS analysis. The retentate contains HLA-I and HLA-I interacting proteins, which were digested, for the analysis of HLA-I abundance and interactome. **b** HLA-I peptide ligand specificity. From the 10 kDa flowthrough fraction, more than 80% of peptides detected were predicted to bind to the JY HLA type (HLA-A*02:01; HLA-B*07:02; HLA-C*07:02). **c** Western blot (WB) detection of HLA-I. HLA-I was detected in the eluate of WCL HLA-I IP, and more strongly in the eluate of EVL HLA-I IP. Full image provided in Supplementary Fig. 9. **d** Mass spectrometry analysis of immuno-purified HLA-I proteins and HLA-I peptide ligands. From EVs, more HLA-I proteins (cumulative MS intensities of HLA-A, HLA-B and HLA-C) and HLA-I peptide ligand species were detected. **e** Peptide length distribution. HLA-I peptide ligands from both whole-cells (WC) and EVs distributed similarly, and expectedly, between 8 to 12 amino acids. **f** Predicted binding affinity. Marginal differences in predicted binding affinity were observed in HLA-I peptides retrieved from WC and EVs. Box plots represent *n* peptides (where *n* has been annotated under each box). The 25%, 50% (median), and 75% quantiles are represented in each box, and the whiskers represent the ±1.5*IQR from the closest quantile. Bar plots represent the total pool of eluted ligands detected in three technical replicates from either WC (green) or EVs (purple).

To compare the HLA-I content and contrast the HLA Class I peptide repertoire in WC and EVs, we performed immunoaffinity purification of HLA-I proteins and HLA class I peptide ligands from JY whole-cell and EV lysates respectively, using the same input load and the same set of immunoaffinity capture steps (Fig. 2a). On acid elution and molecular weight cutoff filtration, HLA class I peptide ligands were separated from HLA-I proteins. In the <10 kDa flowthrough (lower) fraction, HLA class I peptide ligands were recovered for LC-MS/MS analyses. From the >10 kDa retentate (upper) fraction, HLA-I protein and interactors were digested prior to MS analysis. By detailed characterization of both fractions, we aim to compare and contrast both the HLA class I peptide ligand diversity, as well as the interaction network of HLA-I proteins in different spatial compartments.

Performing HLA class I peptide ligandome experiments from a minute amount of EV material required a drastic down-scaling (~20×) of our established workflow^24^, as it was not feasible to obtain milligrams of EVs in contrast to WC. With an adapted high sensitivity workflow, we immunoprecipitated HLA-I proteins from equal input (120 µg) of JY whole-cell lysate or EV lysate. Despite using a much-reduced starting input compared to most other reports of HLA peptide profiling, we did not compromise on purification stringency. As shown in Fig. 2b, more than 80% of all detected ligands were predicted to bind HLA-A*02:01, HLA-B*07:02, or HLA-C*07:02, the defined homozygous HLA type of our JY cell line model. This matches the high ligand purity we typically obtain from HLA class I peptide analyses of WC. As shown in Fig. 2c, more HLA-I proteins were immunoprecipitated from paired load of EV material, suggesting that EVs have much higher HLA content per microgram of protein compared to WC. This was consistent also with the larger number of HLA class I peptide ligands detected from EV material (~3500 vs ~1400 from 120 µg of JY WC, Fig. 2d, right panel), and further corroborated by quantitative proteomics analysis of the 10 kDa filter retentate, where the total intensity of immunoaffinity-captured HLA-I proteins was also higher in EVs (Fig. 2d, left panel). HLA class I peptide ligands from JY WC and EVs distributed similarly in length (Fig. 2e), and were also composed of peptides with similar sequence motifs (Supplementary Fig. 1), albeit a marginally lower nM binding affinity (stronger binding) was observed in EV peptide ligands (Fig. 2f, Supplementary Fig. 2). Taken together, these data reveal that HLA class I peptides on EVs are similar in sequence properties to ligands on WC, except that EVs are more densely inserted with HLA-I complexes.

### EVs preferentially present HLA-B peptide ligands

**Fig. 3:**
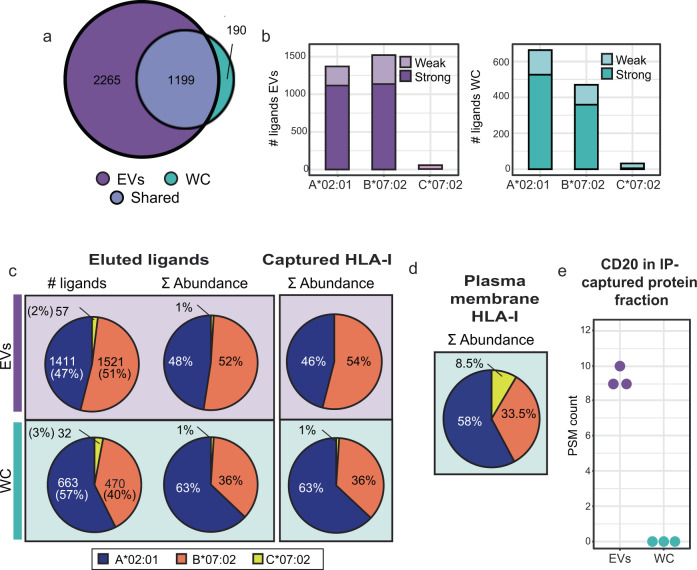
Unique characteristics of the EV HLA-I peptide ligandome. **a** HLA-I peptide species overlap. The JY whole-cell HLA-I peptide ligandome is almost completely sampled in EVs. **b** Predicted peptide binding affinity to JY HLA type. More HLA-B binding peptides were detected from EVs. Distribution of strong (≤50 nM affinity) and weak (≤500 nM affinity) peptide binders respectively. Bar plots represent the total pool of eluted ligands detected in three technical replicates from either WC (green) or EVs (purple). **c** Proportion of HLA-A (blue), HLA-B (orange), HLA-C (yellow) binders. HLA-A binders were over-represented in the JY whole-cell HLA-I peptide ligandome in both peptide species count and summed abundance, which was corroborated by the relative abundance of captured HLA-A and HLA-B proteins. A more balanced proportion of HLA-A to HLA-B was observed in EVs. **d** HLA-I complexes purified from the plasma membrane show a similar abundance distribution to what observed on whole-cells. **e** Spectral count of CD20 co-immunoprecipitated with HLA-I. CD20 (MS4A1) was confidently detected exclusively in the EV HLA-I interactome.

Considering identification overlap in HLA class I peptide ligands, 86% of all the peptides detected from JY WC were also detected in the EV ligandome. In addition, EV HLA peptide profiling revealed another 2265 ligands that were not detected from JY WC (Fig. 3a). This expanded detection may result partially from EVs being more densely loaded with HLA complexes (increased abundance leads to better MS identification), but may also reflect greater substrate peptide diversity arising from the endosomal route during EV biogenesis.

Even though the EV HLA class I peptides appeared similar in sequence properties compared to the JY whole-cell counterpart (Fig. 2), a larger proportion of HLA-B binders was detected from EVs compared to WC (Fig. 3b). From JY WC, HLA-B binders made up 40% of all the ligands detected, whereas the proportion HLA-B binders in EV ligands was substantially higher at 51% (Fig. 3c). From JY WC, the ratio of strong HLA-B binders to strong HLA-A binders is approximately 0.7:1 (Fig. 3b, right panel, dark green), whereas this ratio was almost 1:1 in JY EVs (Fig. 3b, left panel, dark purple). This increased presentation of HLA-B ligands on EVs could also be quantified by a higher summed MS1 intensity (52% in EVs to 36% in JY WC), suggesting that HLA-B binding ligands were also more abundant in EVs (Fig. 3c), in addition to greater sequence diversity (Fig. 3b). This greater quantity and diversity of HLA-B ligands is likely presented on EVs by more HLA-B protein complexes that are loaded into EVs, as HLA-B protein abundance in the immunoaffinity-captured proteins (10 kDa cutoff filter retentate) was also similarly increased compared to HLA-A protein complexes (Fig. 3c, right panel). Although EVs can bud directly from the plasma membrane and contain a big portion of membrane proteins, HLA-B enrichment was not observed when measuring the proteome of plasma membranes derived from JY cells (Fig. 3d), indicating that higher HLA-B presentation is likely an EV-specific trait.

Dominant HLA-B presentation on EVs may also be visualized in an alternative manner. By plotting ranked intensities of all HLA peptides detected in JY EVs against the ranked intensities in WC, we observed that a large number HLA-B ligands were more abundantly detected in JY EVs than WC (Supplementary Fig. 3, orange data points in gray shaded space). This observation further supports the observation that JY EVs present HLA-B complexes more dominantly than WC, and imply that differential loading of HLA-I complexes into EVs may occur and be regulated by still unidentified mechanisms. In search of protein interactors that may regulate preferential EV targeting of HLA-B complexes, we compared the proteins that were co-immunoprecipitated with HLAs. Through analyses of the >10 kDa retentates from both EV and whole-cell HLA pulldowns, CD20 was the only plasma membrane protein that was uniquely and prominently retrieved together with EV HLAs (Fig. 3e). This evidence of interaction may connect CD20 with HLA-B trafficking into the endosomal route. Indeed, a CD20 homolog has been reported to transport receptor tyrosine kinase KIT towards endocytic pathways^33^, which would overlap with the route of EV biogenesis.

### Cysteinylated ligands dominate the PTM-specific EV ligandome

Post-translational modifications (PTMs) on HLA class I peptide ligands have been suggested to affect HLA-I loading affinity as well as the propensity to trigger T cell activation^34,35^. Such properties are highly critical in peptide-based vaccine design and therapeutic efficacy. Particularly for peptide-loaded HLA complexes in the EV, transit through the acidic endosomal compartments, and/or exposure to complex extracellular conditions without redox protection, may already alter the prevalence of background PTMs in comparison to cell surface HLA peptide ligands. Therefore, a clear and comprehensive assessment of modification differences between whole-cell HLA ligandome and EV HLA ligandome is crucial to evaluate if HLA peptide-based vaccines delivered via EVs actually would resemble that of deactivated WC. To our knowledge, such a direct comparison has not been established comprehensively in the field.

**Fig. 4:**
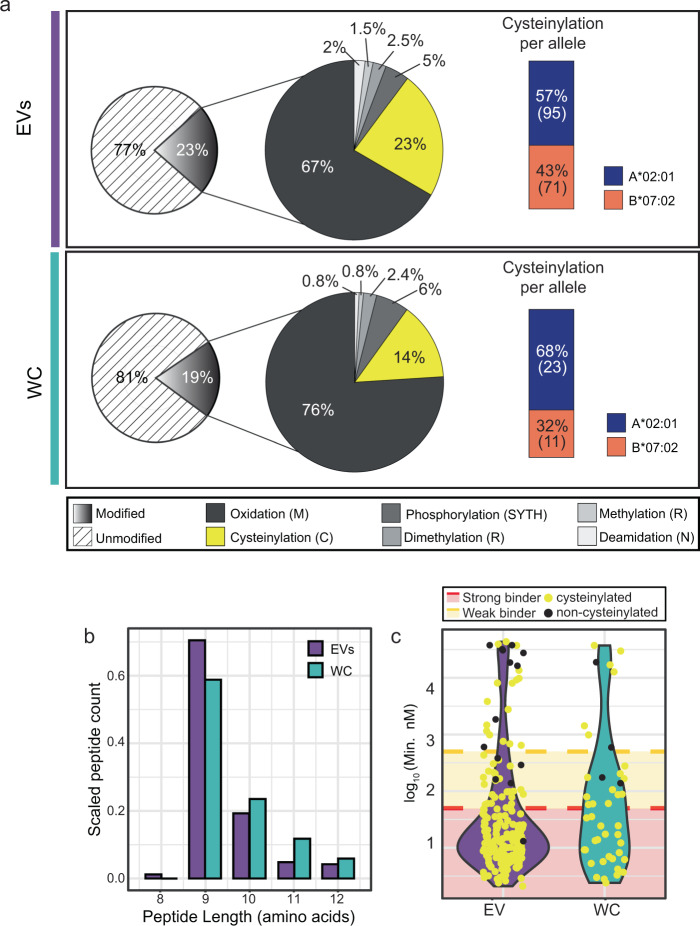
Post-translational modification (PTMs) analysis of EV and whole-cell HLA-I peptide ligands. **a** PTM distribution. EV HLA-I peptide ligands are more frequently cysteinylated than HLA-I peptides from WC. Cysteinylation (yellow) was more abundantly detected on HLA-A binders (blue) than in HLA-B binders (orange). The number of cysteinylated ligands (*n*) derived from each allele is shown in parentheses. **b** Length distribution of the cysteinylated ligands. Y-axis was scaled to total number of peptides identified in three technical replicates from either WC or EVs. **c** Predicted binding affinity (nM) of all cysteine-containing peptides. Cysteinylated cysteine-containing peptides (yellow) were predicted to bind more strongly than non-cysteinylated cysteine-containing peptides.

In our search of PTMs, we did not enrich for specific PTM species, as differential efficiency of the various enrichment steps could skew the relative proportion and intensity with respect to non-modified peptides. Instead, we focused on adding search modifications that have been reported to exist on HLA peptides, and allowing small modifications that are less likely to hinder HLA peptide loading and presentation. As shown in Fig. 4a, both the whole-cell and EV ligandome contain similar proportion of peptides that have one of the six PTMs investigated, but a further breakdown in contribution of different PTMs revealed that only cysteinylation is observed much more frequently on HLA peptides of EV origin (23% vs 14% in JY WC). This is unlikely to be caused by the expanded detection of HLA-B peptide binders from EVs (Fig. 3b), since cysteinylation modifies HLA-A binders more prominently (Fig. 4a). In contrast, the proportion of peptides with any of the other five PTMs did not change noticeably.

Intrigued by the larger proportion of cysteinylated HLA peptides in EVs, we looked further into these peptides. Compared to cysteinylated HLA peptides isolated from JY WC, cysteinylated peptides from EVs appear to be slightly shorter in length (Fig. 4b), and are mostly predicted strong binders (<50 nM predicted binding affinity). Non-cysteinylated cysteine-containing ligands, on the other hand, were rarely predicted to bind to any of the HLA alleles with high affinity in both WC and EVs (Fig. 4c). Interestingly, the distribution of cysteinylation positions also differed slightly between WC and EV ligandomes. The 9-mer cores of the cysteinylated ligands were Gibbs-clustered, and the cysteinylation positions on EV ligands were in general more spread over the non-anchoring residues (Supplementary Figure 4). Collectively, the differences in EV HLA peptide PTMs, as well as PTM position within the peptides, seem to echo that the EV HLA peptide repertoire undergoes a slightly different processing and loading path.

## Discussion

In recent years, EVs are emerging as promising vaccine delivery tools that may even be personalized^21,36^. To date EVs have been mostly pre-loaded artificially with therapeutic agents and RNAs^19,21,36–39^, and the logical next step is to employ EVs as “antigen delivery drones” to the human immune system. In this respect, the systemic distribution of EVs, and wide circulatory contact with other immunity components, also make EVs naturally suitable for presenting short antigen peptides. EVs are not only high-stability formulations^8,40^ in the human blood, but also naturally loaded with an abundance of HLA complexes, as we demonstrate here (Fig. 2c, d).

Compared to pre-loading EVs with a defined peptide antigen, which requires knowledge a priori, harvesting EVs from a source cell line allows the production of a complex EV repertoire with potentially broader immuno-specificities. For instance, a desirable cell line can be maintained and made to produce EVs on demand, akin to an antibody-producing hybridoma^41^. The use of cell line *factories* to “manufacture” human EVs for therapy^19,42,43^ has been attempted. In several phase I/II clinical trials, vaccines delivered by EVs were effective, but much milder than expected^6,44^. A possible explanation could be intrinsic peptide repertoire differences between the cellular and EV-bound ligandomes, such that giving cell-free EVs is not exactly the same as challenging the system with WC. Or alternatively, post-translational modifications on exposed residues (positions 3-–8) of HLA-bound peptide ligands may be modulating immunogenicity^45^ or dampening immune responses^46,47^. Therefore, a comprehensive and detailed comparison of the EV-derived HLA class I ligandome, against the whole-cell counterpart, may help to rationalize the clinical differences in therapeutic efficacy and illuminate the obstacles still impeding better application of EVs in antigen and vaccine delivery. Such an impetus motivated us to pursue this study.

Using the high-sensitivity workflow described herein, we reached an unprecedented identification of ~3500 HLA-I peptide ligands from 120 µg EVs. This gave us ample depth to compare the HLA-I peptide ligandomes between EVs and WC, both in terms of peptide sequences and post-translational modifications. In comparison to the whole-cell HLA-I peptide ligandome, we found that the EV HLA-I peptides were rather similar in length, motif, and sequence properties, in agreement with Synowsky et al.^31^, albeit with a shift towards over-representation of HLA-B binders and peptides with cysteinylated cysteines spanning positions 5 to 8 (Supplementary Fig. 3). This higher proportion of HLA-B ligands agrees well with the higher yield of HLA-B proteins from EVs (Fig. 3c), but the mechanism of additional HLA-B packaging into EVs remains unclear. We hypothesize this may be mediated through CD20, a specific HLA-I interactor^48^, that we detected only in the EV HLA-I interactome (Fig. 3e), but suspect that internalization of specific HLA types along the EV biogenesis route may also be at play. Indeed, differential stability and turnover rate of different HLA allotypes have also been reported previously^49–51^.

In addition to fundamental alterations in the EV HLA-I peptide repertoire due to EV HLA-I sub-composition (specifically HLA-B), the complex biogenesis pathway of EVs may also further modulate the ligand diversity. EVs are formed through multiple routes that involve maturation in the endosomal compartments. It is thus plausible that the distinct environments, and pH, along the EV maturation path may also have an impact on the stability and exchange of HLA-I peptide ligands^52–54^. These may have contributed to the unique EV HLA-I peptide ligand species we identified here (Fig. 3a), as well as the prevalence of modifications on redox-sensitive amino acid residues, for instance cysteine cysteinylation (Fig. 4).

Cysteinylation results from the disulfide linkage of a free cysteine to an unpaired cysteine residue on a protein or peptide. Although free cysteines are inevitably present in the cell culture medium, cysteinylation of HLA-bound peptides has been postulated to also take place endogenously during peptide loading in the endoplasmic reticulum or in cysteine-rich organelles^46^. Therefore, cysteinyl modifications on HLA peptides cannot always be easily brushed aside as artifacts. On the contrary, we still seem to observe higher cysteinylation in EV HLA-I peptide ligands when EVs are in shorter contact-time with cell culture medium in our workflow than WC. In addition, we also did not detect the non-cysteinylated peptide counterpart of any cysteinylated peptides documented here. Taken together, these suggest that the modification may be favored for loading, since 100% complete cysteinylation just from the cell culture medium seemed rather implausible. Outside the scope of this current investigation, experimental structural studies would be of great interest, to address if and how cysteinylation may affect the loading or stability of cysteine-containing peptide ligands inside the HLA-binding pocket.

With a focus on HLA peptide modifications, we quickly faced limitations in assessing or predicting the binding characteristics of post-translationally modified peptides. To date, post-translational modifications on HLA-I peptide ligands have been widely reported^45,55–59^, and from this current study, about 20% of all the HLA-I peptides we detected feature modifications (Fig. 4a). As such the lack of computational solutions to work with these modified peptides is a perennial bottleneck still limiting our understanding of how these may impact peptide loading, half-life, stability, and immunogenicity on a consensus level. On the other hand, the possibility of diverse modifications on every HLA-I peptide also further increases the complexity in the ligandome repertoire, to make systematic profiling all the more challenging.

Nonetheless, considering the major findings presented in this work, EVs indeed appear to be very promising immuno-regulatory therapeutics. EVs not only sample almost the complete whole-cell HLA-I peptide ligandome, but are also more densely loaded with antigen peptides. The source of therapeutic EVs can be renewable, and preserved in the form of a parental cell line, which also allows desired tweaks to be engineered if necessary. Indeed, specific targeting of proteins to the EVs during EV biogenesis is also already achievable by current techniques^60,61^. Here in the JY cell line, we also observed more dominant presentation of HLA-B and HLA-B peptide ligands in the EV. This may be an additional advantage, since HLA-Bs are very polymorphic^62^ with diverse ligand repertoires. It is however critical to note that if the desired antigens to be delivered via EVs contain cysteines, the redox state of these would need to be monitored closely. This is relevant, since cysteinylation has been shown to influence T-cell responses^46,47^.

## Methods

### Cell culture and EV isolation

JY, an Epstein–Barr virus-immortalized cell line homozygous for HLA class I (HLA-A*02:01, HLA-B*07:02, and HLA-C*07:02), was obtained from ATCC, and cultured in RPMI 1640 (Lonza, Switzerland) supplemented with 10% fetal bovine serum (FBS; HyClone, USA), 10 mM L-glutamine, 50 U/mL penicillin and 50 μg/mL streptomycin (Lonza) in a humidified incubator at 37 °C with 5% CO_2_. At a total count of 2e8 cells and <5% cell death, JY cells were washed thrice with PBS and plated at 5e5 cells/mL in secretion media (RPMI 1640 supplemented as previously described but containing 10% Exosome-depleted FBS (ThermoFisher, USA) instead). Conditioned media containing JY-derived EVs was collected after 24 h, spun down at 300 × *g* for 10 min, vacuum filtered on a 0.22 μm Stericup device (Millipore, USA), and kept at 4 °C. Cells were re-plated at 1e6 cells/mL and conditioned media was collected again after 24 h and processed as described above. Pre-cleared media was pooled and immediately processed by ultracentrifugation at 120,000 × *g*, at 4 °C for 2 h in a Sorvall T-865 rotor (ThermoFisher) to pellet EVs. The pellet was thoroughly resuspended in 10 mL PBS to wash away contaminants and purified EVs were finally pelleted again by ultracentrifugation at 120,000 × *g* at 4 °C for 2 h. The EV-pellet was either resuspended in PBS for biophysical and proteome characterization (5%), or lysed separately for immunoaffinity purification of HLA-I complexes and peptide ligands (95%).

### Negative stain electron microscopy (NS-TEM)

Isolated EVs were diluted 1:10 in PBS. Carbon-coated Ted Pella G400 copper grids were glow discharged, and immediately incubated with 5 μL the diluted EVs for 30 s. Excess solution was blotted away, and the specimens were stained twice with 5 μL of 2% (w/v) of uranyl acetate each. NS-TEM data was collected on a Tecnai 12 transmission electron microscope (FEI, Switzerland) equipped with a SIS CCD MegaView II camera. Images were acquired with a pixel size of 6.5 Å/pixel at a defocus of −10 to −15 μm.

### Nanoparticle tracking analysis (NTA)

Isolated EVs were diluted 1:1000 in PBS to a final volume of 1 ml to be analyzed in a NanoSight NS500 (Malvern Panalytica, UK), equipped with a sCMOS camera and a Blue405 laser. For the recordings, the camera level was set to 16. The sample concentrations were ~50 particles/frame, in the range of optimal operation between 30 and 100 particles/frame. Four videos of 1 min were taken at 25 FPS and averaged with the built-in NanoSight Software NTA v.3.3 using a detection threshold of 5.

### EV and whole-cell lysis

JY EVs and WC were lysed on an end-to-end rotating platform at 4 °C in Pierce IP Lysis Buffer (ThermoFisher) supplemented with 1× complete protease inhibitors (Sigma-Aldrich,USA), 50 μg/mL DNase Ι (Sigma-Aldrich), and 50 μg/mL RNase A (Sigma-Aldrich). EVs were lysed in 200 μL of lysis buffer for 30 min followed by 20 cycles of sonication at 4 °C (30 s on, 30 s off) in a Bioruptor (Diagenode, Belgium). Cells were lysed in 10 mL of lysis buffer for 1 h. The lysates were spun down at 20,000 *g* at 4 °C for 1 h and the aqueous phase containing the solubilized proteins was kept. Lysate protein concentrations were determined with a microplate BCA-protein assay (ThermoFisher). Aliquots of 15 μg were taken for further characterization and all the samples were snap-frozen until further processing.

### SDS-page and western blot

EV and WC lysates, and 10 kDa filtration retentates were resolved on 12% Bis-Tris Criterion XT precast gels (Biorad, USA) with 1x XT-MOPS buffer at fixed voltage of 150 V for about 2 h. Proteins were stained in-gel using Imperial Protein Stain (ThermoFisher). For Western detection, proteins were transferred to a PVDF membrane in Towbin buffer (0.025 M Tris, 0.192 M glycine, 20% methanol) at 100 V and 4 °C for 1 h. Membranes were washed 3× with TBS buffer containing 1% Tween-20 (TBST) and then blocked for 1 h in TBST supplemented with 5% Blotting-Grade Blocker (Biorad). Primary antibodies (W6/32 at 1:5,000 or α-CD81 at 1:200) were incubated at 4 °C overnight in TBST supplemented with 1% milk. Secondary antibody incubations were done using HRP conjugated α-mouse IgG antibody (1:2000 dilution) for 2 h at room temperature in the same buffer. Between and after antibody incubations, membranes were washed 3 × 10 min in TBST. HRP signal was visualized using SuperSignal West Dura (ThermoFisher) substrate, on an Amersham Imager 600 (GE healthcare, USA).

### Immuno-affinity purification of HLA complexes and peptide ligands

To capture class I HLA complexes from EV and whole-cell lysates, 5 µg of W6/32 pan-HLA class I antibody was coupled and cross-linked to 5 µL protein A/G beads and incubated with 120 µg of lysate overnight at 4 °C in an end-to-end rotating platform. The beads were washed four times with 100 µL of cold PBS and HLA complexes were eluted with cold 10% acetic acid. We have tested that W6/32 is specific for human HLA molecules, and does not cross-react with Bovine MHC (BoLa), which may still be present in trace amounts in EV-depleted FBS (Supplementary Fig. 5).To maximize the retrieval of HLA complexes, lysates were re-loaded twice into the beads and the same procedure was repeated. Elutions were pooled and peptide ligands were separated from HLA complexes by ultrafiltration at 12,000 *g* in 10 kDa molecular weight cutoff filters (Millipore). The flow-through, containing the peptide ligands, was desalted using Pierce C18 10 µL bed Stage tips (ThermoFisher), vacuum dried and stored at −20 °C before LC-MS analysis. Proteins in the retentate fraction were recovered by solubilizing in 8 M Urea/50 mM ammonium bicarbonate.

### Plasma membrane fractionation

JY Plasma membranes were enriched using a membrane fractionation kit (ab 65400, Abcam, Cambridge, UK) as described previously^63^. Washed cell pellets were gently resuspended in ice-cold isotonic and detergent-free homogenization buffer supplemented with 1× cOmplete protease inhibitors, 50 μg/mL DNase I, and 50 μg/mL RNase A. Cells were disrupted manually at 4 °C with a hand-held glass homogenizer. Any remaining intact cells were pelleted at 700 *g*, for 10 min and discarded. Total membranes were pelleted from the supernatant at 10,000 *g*, for 30 min, and further partitioned in partially miscible gradients established by mixing 200 μl each of the upper and lower phase solutions (Abcam ab 65400). The upper phase, containing plasma membrane proteins, was collected and precipitated for 30 min at 4 °C by 5 times dilution with ultra-pure water. Plasma membrane proteins were then pelleted at 20,000 *g* for 30 min, resuspended in 8 M Urea, 50 mM Ammonium bicarbonate, 0.2% Sodium deoxycholate, and then digested as described hereafter.

### Protein digestion

To perform proteomics characterization of WC and EV lysates, 5 μg of total proteins from EVs and WC were digested as follows. Each sample was diluted to a final 4 M Urea/50 mM ammonium bicarbonate and proteins were reduced in 4 mM dithiothreitol (DTT) for 1 h, and then cysteine-alkylated in 8 mM iodoacetamide for 30 min in the dark. The alkylation reaction was quenched by further addition of 4 mM DTT, and proteins were pre-digested by Lys-C (1:50 enzyme-protein ratio) at 37 °C for 4 h. Samples were diluted further to 1 M Urea/50 mM ammonium bicarbonate and digested overnight at 37 °C by Trypsin (Promega, USA) at 1:50. Peptides were acidified to 5% Formic acid and were desalted using Pierce C18 10 µL bed Stage tips (ThermoFisher). Peptides were vacuum-dried and stored at −20 °C before LC-MS analysis. The same procedure was applied for digestion of 10 kDa retentates.

### LC-MS/MS

All samples were analyzed in an UHPLC 1290 system (Agilent, USA) coupled to an Orbitrap HF-X mass spectrometer (Thermo Scientific, USA). Peptides were trapped (Dr. Maisch Reprosil-Pur C18-AQ, 3 µm, 2.5 cm × 100 µm) for 5 min in solvent A (0.1% Formic acid in water) and then separated on an analytical column (Agilent Poroshell, 120 EC-C18, 2.7 µm, 50 cm × 75 µm) using a linear gradient of solvent B (0.1% Formic acid in 80% acetonitrile). For HLA peptide ligand analyses, a gradient of 7–40 % B in 90 min was used. For lysate proteomics, a gradient of 13–44% B in 95 min. Finally, 10 kDa retentate analysis, a gradient of 13–40% B in 65 min was used.

The mass spectrometer was operated in data-dependent mode, at a resolution of 60,000 for MS1 and 30,000 for MS2. HLA peptide data were acquired at 400–650 m/z and precursor ions were accumulated for 50 ms or until a AGC target value of 3e6 was reached. The 15 most abundant doubly and triply charged precursors were selected for fragmentation, after accumulation to the AGC target value of 1e5 within 250 ms. HCD fragmentation was performed at 27% NCE. Dynamic exclusion time was set to 16 s. Proteomics data were acquired at 375–1600 m/z, with precursor accumulation of up to 3e6 AGC target value within 20 ms. The 15 most abundant ions (with charge states 2 to 5) were selected for HCD fragmentation, after accumulation to the AGC target value of 1e5 within 50 ms. Dynamic exclusion times were 16 s and 12 s, for 95 min and 65 min gradients respectively. From all paired samples used in this study (WC and EV ligandomes and proteomes), three technical replicates were measured by LC-MS/MS.

### Database search

For HLA peptide analyses, raw data were searched in Proteome Discoverer (v_2.3, Thermo Scientific) using Sequest HT against the SwissProt human database (downloaded on 09/2019, containing 20,431 protein sequences), curated to match JY cells HLA alleles (HLA-A*02:01, HLA-B*07:02 and HLA-C*07:02) and appended with common FBS contaminants. Unspecific searches were performed for precursors with mass between 797 and 1950 Da, corresponding to 8–12 amino acid peptides. Precursor ion and fragment ion tolerance were set to 10 ppm and 0.02 Da respectively, and methionine oxidation and cysteine cysteinylation were set as variable modifications for the main search. For modification-specific analyses, searches for arginine (di-)methylation, phosphorylation (S/T/Y/H) and asparagine deamidation were performed separately always including methionine oxidation. Identified peptides were filtered to 5% false discovery rate (FDR) using percolator algorithm^64^.

For proteomics analysis, raw data were searched in MaxQuant (v_1.6.10.4.3)^65^ against the same database using the Andromeda search engine. In this case, trypsin was set as the digestion enzyme and up to two missed cleavages were allowed. Carbamidomethylation of cysteines was set as a fixed modification while protein N-terminal acetylation and methionine oxidation were set as variable modifications. Label-free quantification (LFQ) was enabled using a minimum ratio count of two and both razor and unique peptides for quantification. Precursor ion tolerance was set to 20 ppm for the first search and 4.5 ppm after recalibration, and fragment ions tolerance was set to 20 ppm. FDR of 1% was set at both PSM and protein level by using a reverse decoy database strategy.

### Data analysis

Immunopeptidomics data were analyzed using Excel and in-house built R scripts^66^, and refined with Illustrator 2020 (Adobe, USA). Binding affinity (BA) predictions were performed with NetMHC 4.0^67^ against the JY HLA type (HLA-A*02:01, HLA-B*07:02 and HLA-C*07:02). Predicted BAs were further curated manually to achieve a stringing binder cut-off. Peptide ligands with BA ≤ 50 mM, 50 < BA ≤ 500 or BA > 500 to HLA proteins were considered strong, weak or non-binders respectively. For cysteinylation binding affinity predictions, cysteine residues of cysteinylated peptides were substituted to “X” since algorithms cannot currently predict modified amino acids. Sequence logos were produced with Seq2Logo using the (P- weighted) Kullback-Leibler method^68^ and only the NetMHC4.0 calculated 9-mer cores were used for these plots. For quantitative analysis of the ligandomes, data were processed using the Prostar software^69^ and normalization of EV and WC ligandomes was achieved by mean-centering approach. Subsequently, ranks were calculated from peptides shared between EV and WC ligandomes using normalized abundance values.

All proteomics data were analyzed using Perseus software (v_1.6.14)^70^. Proteins quantified (LFQ) in two out of three replicates in EV or WC lysates or in the >10 kDa captured fractions were log_2_ transformed and missing values were replaced individually for each sample from the normal distribution. Statistical differences were assessed by a two-sided Student’s *T* test and corrected *p* values (*q* value) were calculated using the permutation method with up to 250 iterations. Proteins were considered significant when *q* value ≤ 0.05 and fold change ≥ 2. All plots were generated using R packages and refined with Illustrator 2020.

### Statistics and reproducibility

All proteomics measurements were performed in technical triplicates, as indicated in the respective figure legends. For comparisons of HLA peptide proportion, identifications from three injection replicates were summed in a non-redundant manner per HLA allele. All experiments have been replicated and are reproducible.

### Reporting summary

Further information on research design is available in the Nature Research Reporting Summary linked to this article.

## Supplementary information

Supplementary Information

Description of Supplementary Files

Supplementary Data 1

Reporting Summary

## Data Availability

Proteomics and immunopeptidomics raw data have been deposited to ProteomeXchange Consortium via the PRIDE repository^71^ and can be accessed through the identifier PXD021177. Source data are provided with this paper. All Uncropped and unedited blot and gel images for Figs. 1c–e, 2c, Supplementary Figure 5 are provided in the Supplementary Figs. 6–10. All data for visualization are provided in Supplementary Data 1.
