# Supplementary material for: HLA-B and cysteinylated ligands distinguish the antigen presentation landscape of extracellular vesicles
Source: Commun Biol. 2021 Jul 1;4:825. doi: 10.1038/s42003-021-02364-y (PMC8249458; doi:10.1038/s42003-021-02364-y)
Supplement: Supplementary file 1 — Supplementary Information [file 42003_2021_2364_MOESM1_ESM.pdf]

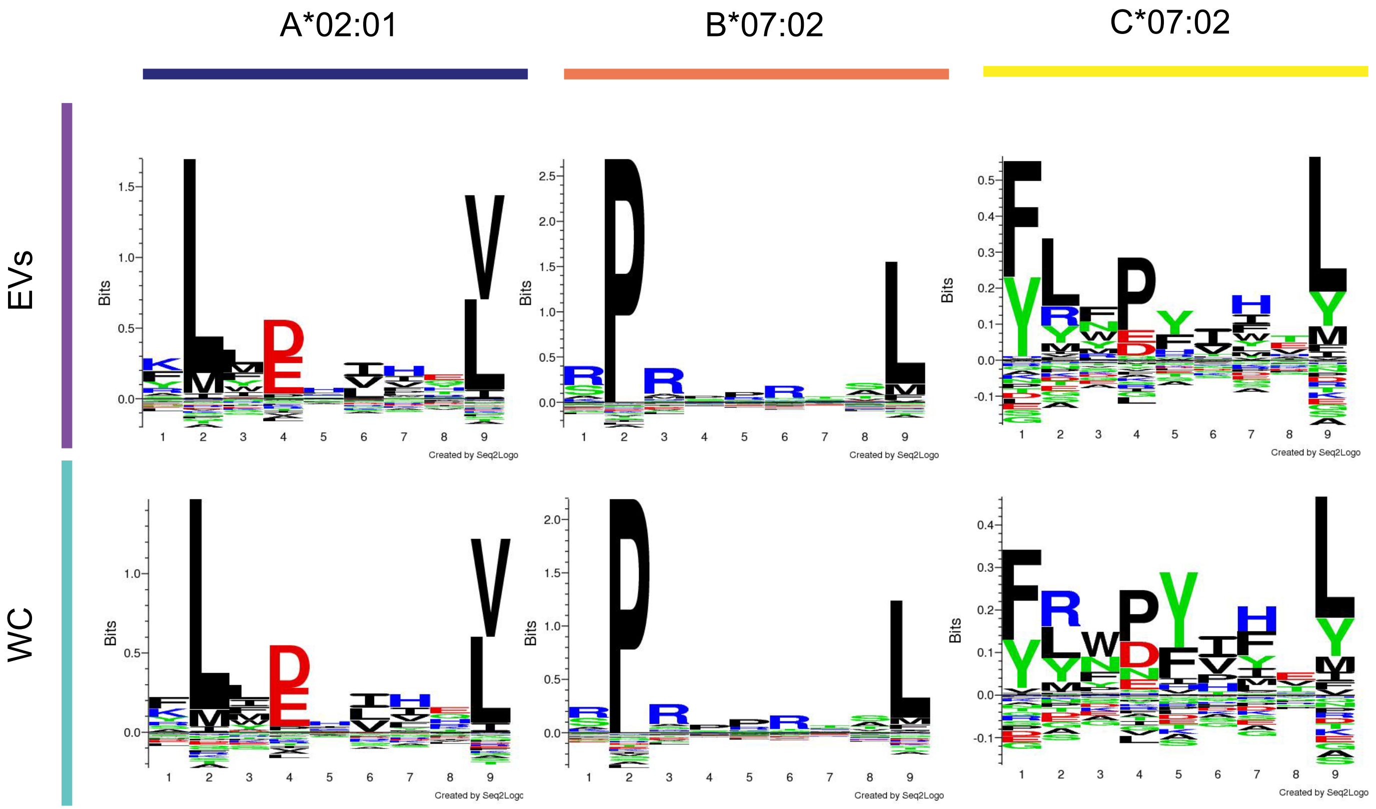

Supplementary Figure 1. Allele-specific sequence motifs of EV- and WC-eluted HLA-I peptide ligands. The predicted 9-mer cores (from NetMHC 4.0) for HLA-A*02:01 (blue), B-07:02 (orange) and C*07:02 (yellow) eluted ligands are visualised by Gibbs clustering, and the empirical HLA-I peptide sequence motifs are displayed. HLA-I peptides eluted from WC (whole-cells, green) and EVs (purple) were similar in sequence motifs.

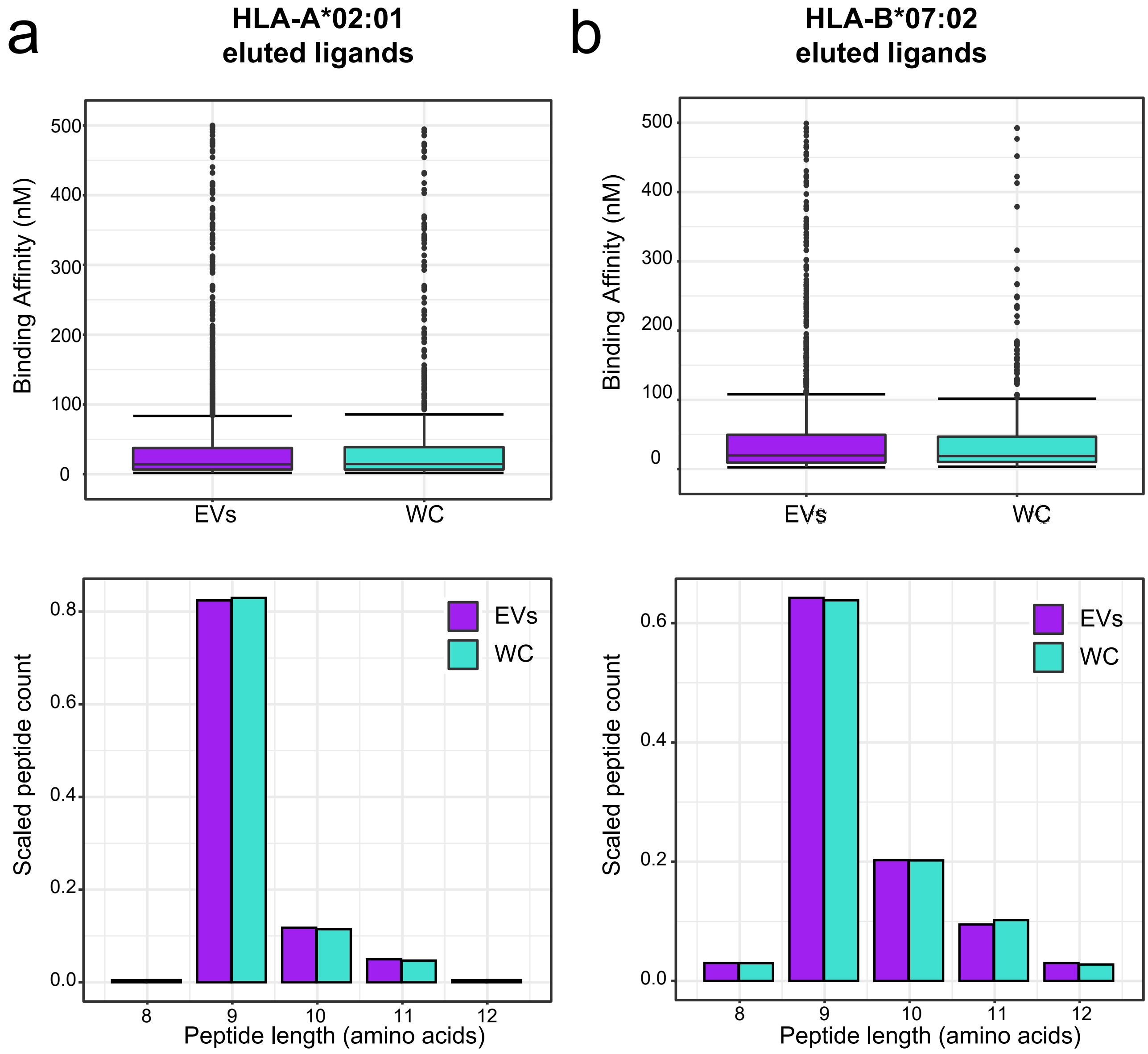

Supplementary Figure 2. Predicted binding affinity and peptide length distribution per HLA type. (a) Predicted binding affinity and length distribution of HLA-A*02:01 ligands eluted from EVs or whole-cells. (b) Predicted binding affinity and length distribution of HLA-B*07:02 ligands eluted from EVs (purple) or whole-cells (WC, green). Box plots represent peptides where the 25%, 50% (median) and 75% quantiles are represented in each box, and the wishkers represent the ±1.5*IQR from the closest quantile.

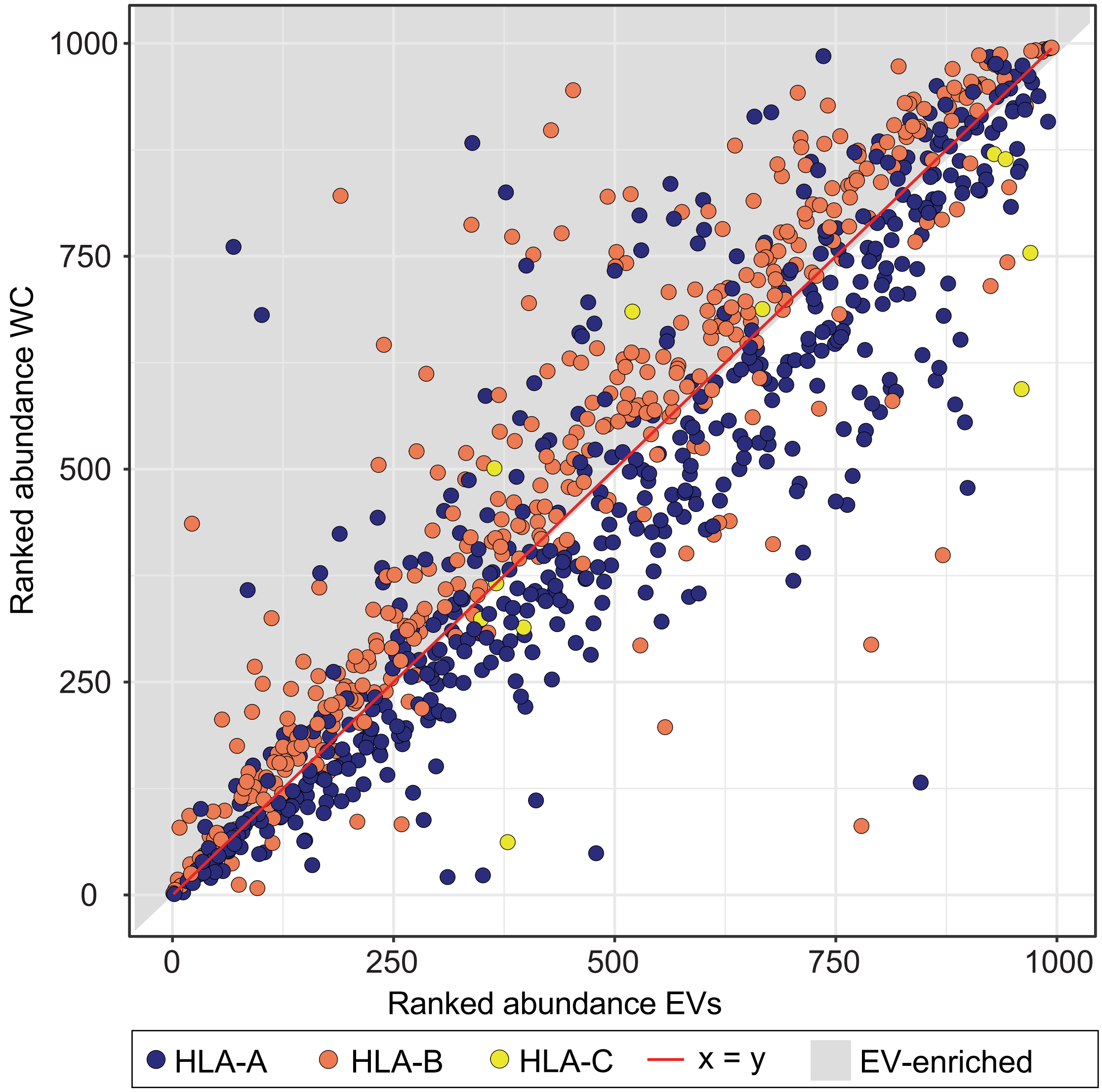

Supplementary Figure 3. Ranked abundance of HLA-I peptide ligands detected in both WC and EV ligandomes. Abundance of shared ligands between EVs and whole-cells (WC) were ranked in decreasing order (highest abundance as rank 1). Red line indicates no difference in rank. Grey shaded area marks ligands with abundance-rank higher in EVs than in WC. Over-representation of HLA-B binders (orange dots) in the grey space indicates many HLA-B binders were more abundantly detected in EVs. HLA-A derived ligands are represented by blue dots, and HLA-C ligands are represented by yellow dots.

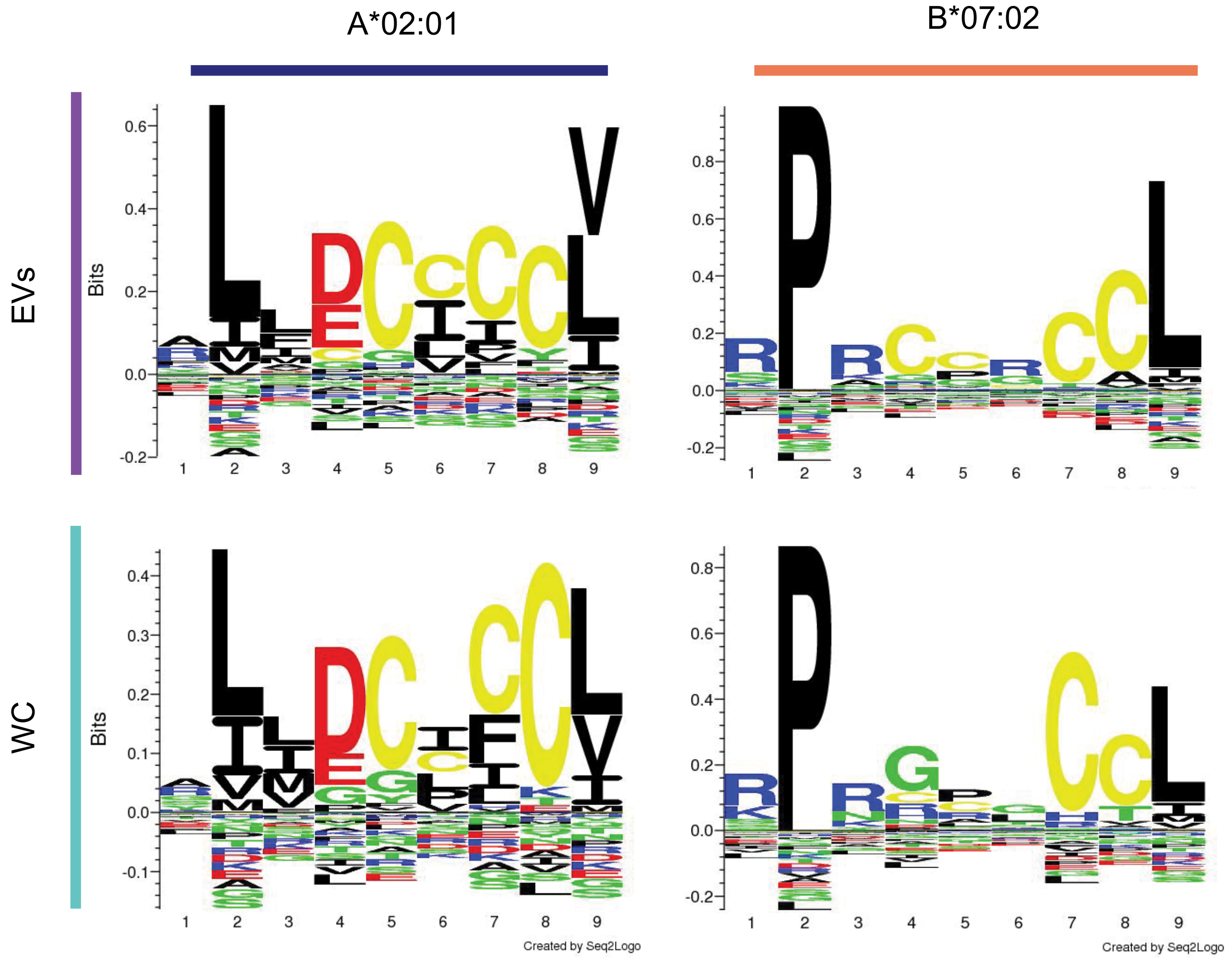

Supplementary Figure 4. Cysteinylation positions on HLA-I peptide ligands. Cysteinylation positions were localised in the predicted 9-mer cores (from NetMHC 4.0) and visualised by Gibbs clustering. All peptides detected contain only 1 cysteine residue and the plot represents the frequency of localization of the cysteinylated cysteine. Clustering shows that cysteinylation occurs most frequently at the C-terminal part of the cysteine-containing peptides in positions 5 to 8 of the ligands.

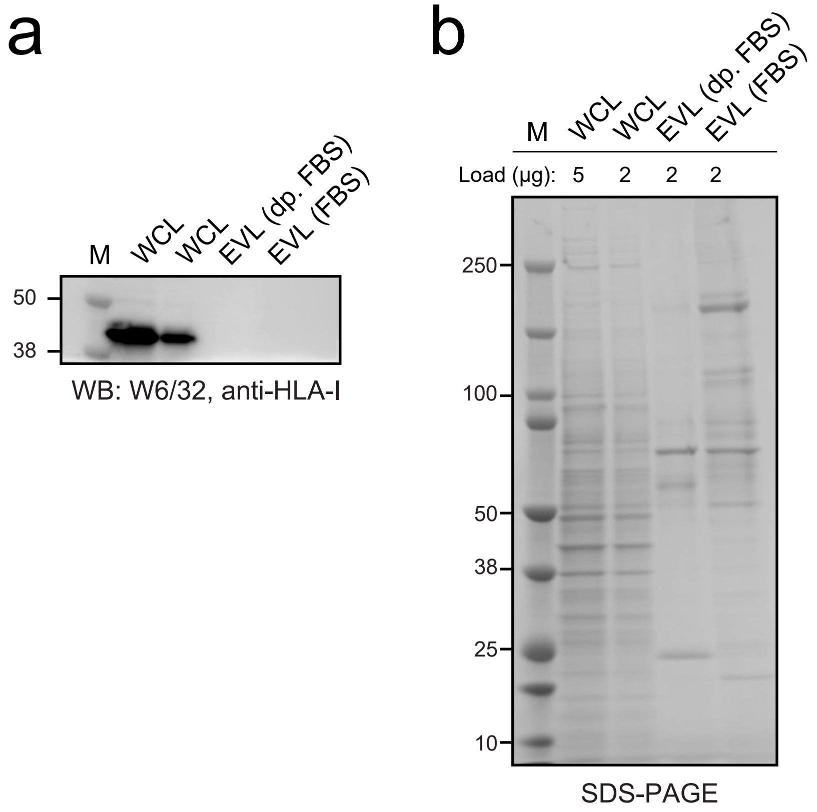

Supplementary Figure 5. W6/32 antibody does not cross-react to bovine MHC (BoLA). (a) Western blot detection performed on 5 and 2 micrograms of JY whole-cell lysates (WCL), and 2 micrograms of EV lysates (EVL), isolated from either EV-depleted fetal bovine serum (dp. FBS) or regular FBS. W6/32 antibody binds specifically to human HLA, but not bovine MHC (BoLA). To obtain bovine EVs, 20 mL of either the depleted FBS used in this study or normal FBS were diluted to 20% in raw RPMI-1640 and EVs were pelleted by ultracentrifugation as described in Materials and Methods. Full image provided in Supplementary Figure 10. **(b)** SDS-PAGE showing the protein profiles of all samples.

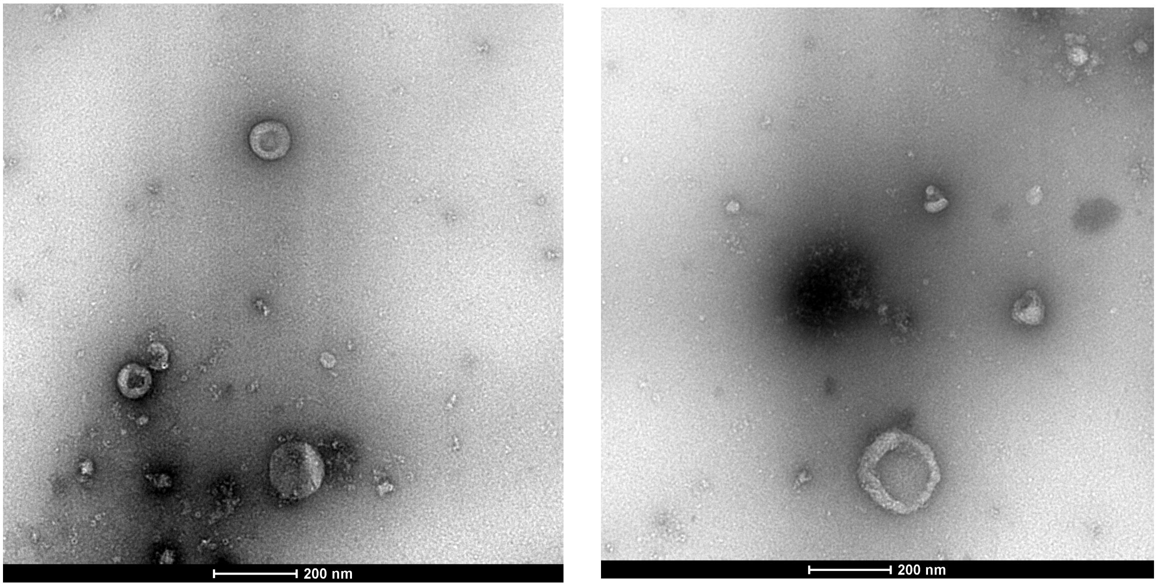

Supplementary Figure 6. Raw negative stain transmission electron microscopy (NS-TEM) scans. NS-TEM analysis of the isolated extracellular vesicles (EVs) shown in Figure 1c. Morphology of the isolated EVs were cup-shaped. Scale bars: 200 nm.

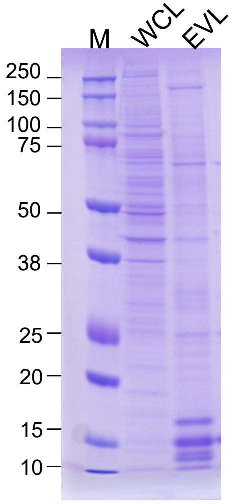

Supplementary Figure 7. Unedited SDS-PAGE image of whole-cell (WCL) and extracellular vesicle lysate (EVL) material. SDS-PAGE analysis shown Figure 1d. Dominant protein bands in WCL and EVL were mutually exclusive. M: molecular weight marker.

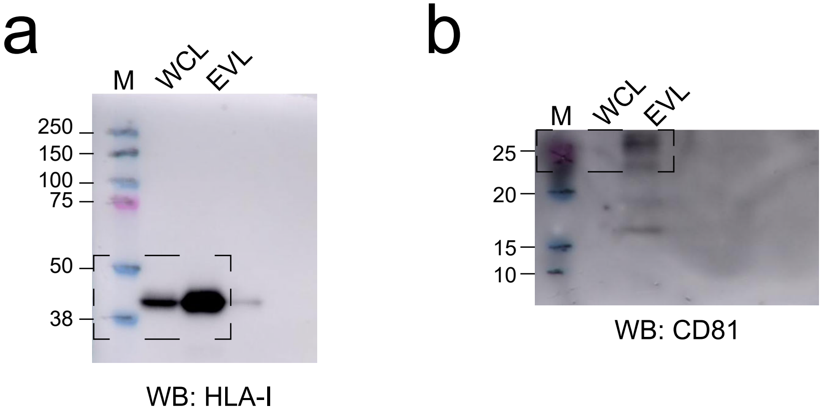

Supplementary Figure 8. Uncropped western blot images for HLA-I and CD81 detection from whole-cell (WCL) and extracellular vesicle lysate (EVL) material. (a) The upper part of the membrane was blotted against HLA-I and **(b)** the lower part of the membrane was blotted against CD81. HLA-I and CD81 western blot. From 5 μg load of EVL and WCL, prominent enrichment of HLA-I and CD81 were observed in EVL.The areas shown in Figure 1e are highlighted with a dotted line. M: molecular weight marker.

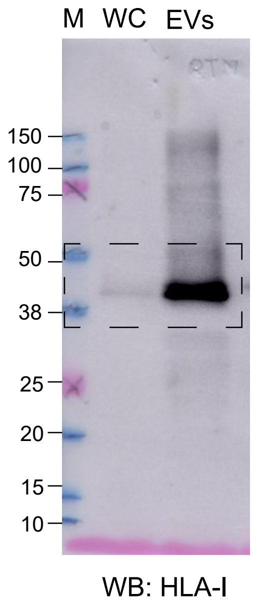

Supplementary Figure 9. Uncropped western blot images for HLA-I detection from the captured protein fraction of whole-cells (WC) or extracellular vesicles (EVs) IP eluates. The membrane was blotted against HLA-I. HLA-I was detected in the eluate of WCL HLA-I IP, and more strongly in the eluate of EVL HLA-I IP.The area shown in Figure 2c is highlighted with a dotted line. M: molecular weight marker.

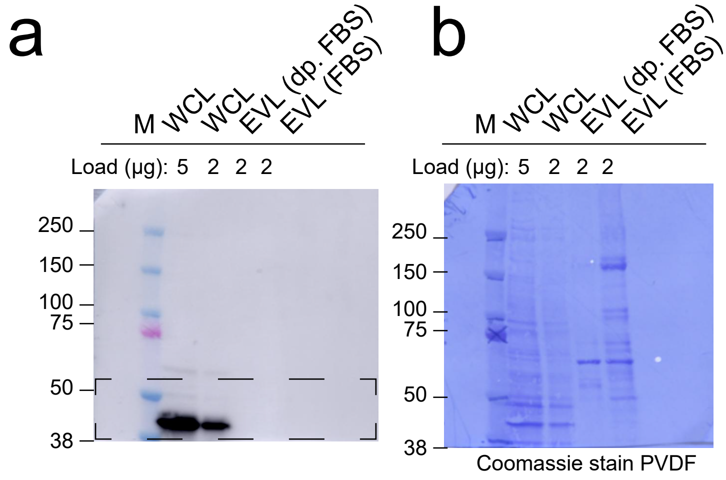

Supplementary Figure 10. Uncropped western blot images for HLA-I detection from human and bovine samples. (a) Western blot detection performed on upper membrane for 5 and 2 micrograms of JY whole-cell lysates (WCL), and 2 micrograms of EV lysates (EVL), isolated from either EV-depleted fetal bovine serum (dp. FBS) or regular FBS. W6/32 antibody binds specifically to human HLA, but not bovine MHC (BoLA). The area shown in Supplementary Figure 5a is highlighted with a dotted line. **(b)** Coomassie stain of the PVDF membrane. M: molecular weight marker.
