# Supplementary material for: HLA-B and cysteinylated ligands distinguish the antigen presentation landscape of extracellular vesicles
Source: Commun Biol. 2021 Jul 1;4:825. doi: 10.1038/s42003-021-02364-y (PMC8249458; doi:10.1038/s42003-021-02364-y)
Supplement: Supplementary file 2 — Description of Supplementary Files [file 42003_2021_2364_MOESM2_ESM.pdf]

Description of Additional Supplementary Files

File name: Supplementary Data 1
Description:
Sheet 1: *Figure 1B:* NTA Experiment Summary File
Sheet 2: *Figure 1C:* Raw NS-TEM Scans
Sheet 3: *Figure 1D:* SDS-Page of WCL and EVL. Samples were run in 12% Bis-Tris Criterion gels (Biorad), usin the molecular weight marker (M): Dual Color (Biorad). WCL: whole cell lysate, EVL: extracellular vesicle lysate.
Sheet 4: *Figure 1E:* Western blot analysis of WCL and EVL. Samples were run in 12% Bis-Tris Criterion gels (Biorad) and transferred to PVDF membranes. The molecular weight marker (M) used is Dual Color (Biorad). WCL: whole cell lysate, EVL: extracellular vesicle lysate.
Sheet 5: *Figure 1F:* EV and WC makers. Log2 transformed protein abundances are shown as measured from WCL and EVL proteomes. For RABs, Integrins (ITGs) and Annexins (ANXAs) protein families, the mean of all protein members was taken.
Sheet 6: *Figure 1G:* Volcano Plot - EV vs WC proteome. Log2 transformed protein abundances were averaged for WCL and EVL
proteomes. The log2 fold change (fc) was calculated by subtracting EVL and WCL mean abundances. Student T-test p-values are reported and were -log10 transformed for visualization.
Sheet 7: *Figure 2B:* Specificity. Proportion of binders (Minimum nM affinity ≤500) and non-binders observed for peptides eluted from EVs(purple) or WCs (green) is shown (top-left table). Criteria for filtering is shown (top-right table). Binding affinity (nM) values are provided on the bottom table for each HLA allele (HLA-A, blue; HLA-B, orange, and HLA-C, yellow). Affinity predictions were obtained from NetMHC 4.0.
Sheet 8: *Figure 2C:* Western blot analysis against HLA-I of captured protein fractions derived from EVs or WC. Samples were run in 12% Bis-Tris Criterion gels (Biorad) and transferred to PVDF membranes. The molecular weight marker (M) used is Dual Color (Biorad). WCL: whole cell lysate, EVL: extracellular vesicle lysate.
Sheet 9: *Figure 2D:* Mean summed abundance (HLA-A, B and C) in captured protein fraction, and total # of non-redundant peptides eluted from EVs and WC.
Sheet 10: *Figure 2E and 2F:* Length distribution and Binding affinity per length. Source (EV, extracellular vesicle or WC, whole-cells), peptide sequence, peptide lengths and minimum nM affinity are reported, together with a categorical column providing allele-specific information of the binders (HLA-A, blue; HLA-B, orange, and HLA-C, yellow). Affinity predictions were obtained from NetMHC 4.0.
Sheet 11: *Figure 3A:* Venn Diagram. Total peptide sequences identified for EVs (purple) or WC (green) are shown.
Sheet 12: *Figure 3B:* Ligands per allele. A table is shown including the peptide sequence for either WC (green) or EVs (purple), and the nM affinity for each allele (HLA-A, blue; HLA-B, orange, and HLA-C, yellow). Criteria for filtering is shown in the top-right note. Affinity predictions were obtained from NetMHC 4.0.
Sheet 13: *Figure 3C, 3D:* Pie charts. The frequency of the indicated field is reported for

each allele (HLA-A, blue; HLA-B, orange, and HLA-C, yellow).
Sheet 14: *Figure 3E:* CD20 in IP captured protein fraction. MS4A1 (CD20) PSM count is reported for each technical replicate of the proteins co-eluting with HLA-I in the IP, as measured in the capture protein fraction derived from either WC, whole cells; or EV, Extracellular vesicles.
Sheet 15: *Figure 4A:* Modified peptides. Frequencies of modified and unmodified peptides are shown in the first table (upper). Frequencies of specific modifications are shown in the second table. Cysteinylated peptide count per allele is shown in the third table (lower). WC, whole-cells; EV, extracellular-vesicles. (HLA-A, blue; HLA-B, orange, and HLA-C, yellow).
Sheet 16: *Figure 4B:* Length of cysteinylated peptides. Peptide sequences and their length are provided, where the cysteinylated cysteine residue has been substituted by X. WC, whole-cells; EV, extracellular vesicles.
Sheet 17: *Figure 4C:* Figure 4C: Cysteine-containing peptides, Binding Affinity. Peptide sequences, their nM affinity per allele as well as the predicted 9-mer cores are shown. Cysteinylated cysteines have been substituted by X. HLA-A, blue; HLA-B, orange, and HLA-C, yellow. WC, whole-cells; EV, extracellular vesicles. Affinity predictions were obtained from NetMHC 4.0.
Sheet 18: *Supplementary Figure 1:* All ligands, 9-mer cores for Motif Analysis. Peptide sequences, their nM affinity per allele as well as the predicted 9-mer cores are shown. Cysteinylated cysteines have been substituted by X. HLA-A, blue; HLA-B, orange, and HLA-C, yellow. WC, whole-cells, green; EV, extracellular vesicles, purple. Affinity predictions were obtained from NetMHC 4.0. The 9-mer cores were used for motif analysis.
Sheet 19: *Supplementary Figure 2:* Binding affinity and Length of ligands per allele. Source (EV, extracellular vesicle or WC, whole-cells), peptide sequence, peptide lengths and minimum nM affinity are reported, together with a categorical column providing allele-specific information of the binders (HLA-A, blue; HLA-B, orange and HLA-C, yellow). Affinity predictions were obtained from NetMHC 4.0.
Sheet 20: *Supplementary Figure 3:* Ranked ligand abundance in EV and WC ligandome. Normalized ligand abundance (mean-centering) of common ligands (EVs and WCs) were averaged for either EV or WC and ranked, where rank=1 is the most abundant ligand. Ranks are provided for EVs(purple) and WC (green) and the allele to which peptides bind is annotated in blue (HLA-A), orange (HLA-B) or yellow (HLA-C).
Sheet 21: *Supplementary Figure 4:* Cysteinylated ligands, 9-mer cores for Motif Analysis. The predicted 9-mer cores are shown for WC, whole-cells, green; and EV, extracellular vesicles, purple. The 9-mer cores were obtained from NetMHC 4.0 and were used for motif analysis.
Sheet 22: *Supplementary Figure 5:* SDS-page and western blot nalysis against MHC-I of either human or bovine source. W6/32 (anti-HLA) antibody does not cross-react bovine derived EVs. Upper membrane and SDS-page raw results are shown. A legend is provided containing sample names. Samples were run in 12% Bis-Tris Criterion gels (Biorad), using the molecular weight marker (M): Dual Color (Biorad). WCL: whole cell lysate, EVL: extracellular vesicle lysate.
