# Supplementary material for: HLA-B and cysteinylated ligands distinguish the antigen presentation landscape of extracellular vesicles
Source: Commun Biol. 2021 Jul 1;4:825. doi: 10.1038/s42003-021-02364-y (PMC8249458; doi:10.1038/s42003-021-02364-y)
Supplement: Supplementary file 4 — Reporting Summary [file 42003_2021_2364_MOESM4_ESM.pdf]

nature research

Corresponding author(s): Wei Wu

Last updated by author(s): May 20, 2021

Reporting Summary

Nature Research wishes to improve the reproducibility of the work that we publish. This form provides structure for consistency and transparency in reporting. For further information on Nature Research policies, see our Editorial Policies and the Editorial Policy Checklist.

Statistics

For all statistical analyses, confirm that the following items are present in the figure legend, table legend, main text, or Methods section.

n/a	Confirmed	
☐	☒	The exact sample size (*n*) for each experimental group/condition, given as a discrete number and unit of measurement
☐	☒	A statement on whether measurements were taken from distinct samples or whether the same sample was measured repeatedly
☐	☒	The statistical test(s) used AND whether they are one- or two-sided<br>*Only common tests should be described solely by name; describe more complex techniques in the Methods section.*
☒	☐	A description of all covariates tested
☐	☒	A description of any assumptions or corrections, such as tests of normality and adjustment for multiple comparisons
☐	☒	A full description of the statistical parameters including central tendency (e.g. means) or other basic estimates (e.g. regression coefficient) AND variation (e.g. standard deviation) or associated estimates of uncertainty (e.g. confidence intervals)
☐	☒	For null hypothesis testing, the test statistic (e.g. *F*, *t*, *r*) with confidence intervals, effect sizes, degrees of freedom and *P* value noted<br>*Give P values as exact values whenever suitable.*
☒	☐	For Bayesian analysis, information on the choice of priors and Markov chain Monte Carlo settings
☒	☐	For hierarchical and complex designs, identification of the appropriate level for tests and full reporting of outcomes
☒	☐	Estimates of effect sizes (e.g. Cohen's *d*, Pearson's *r*), indicating how they were calculated

Our web collection on statistics for biologists contains articles on many of the points above.

Software and code

Policy information about availability of computer code

Data collection	Orbitrap Tune (Thermo Scientific); XCalibur
Data analysis	MaxQuant version 1.6.7; Perseus version 1.6.14; Proteome Discoverer version 2.4; NetMHC 4.0; Prostar 2.0; homemade R scripts using common libraries for plotting (R version 4.03)

For manuscripts utilizing custom algorithms or software that are central to the research but not yet described in published literature, software must be made available to editors and reviewers. We strongly encourage code deposition in a community repository (e.g. GitHub). See the Nature Research guidelines for submitting code & software for further information.

Data

Policy information about availability of data

All manuscripts must include a data availability statement. This statement should provide the following information, where applicable:

- Accession codes, unique identifiers, or web links for publicly available datasets
- A list of figures that have associated raw data
- A description of any restrictions on data availability

Proteomics and immunopeptidomics raw data have been deposited to ProteomeXchange Consortium via the PRIDE repository71 and can be accessed through the identifier PXD021177. Source data are provided with this paper.

Field-specific reporting

Please select the one below that is the best fit for your research. If you are not sure, read the appropriate sections before making your selection.

☒ Life sciences ☐ Behavioural & social sciences ☐ Ecological, evolutionary & environmental sciences

For a reference copy of the document with all sections, see nature.com/documents/nr-reporting-summary-flat.pdf

Life sciences study design

All studies must disclose on these points even when the disclosure is negative.

Sample size	This study features paired comparisons of cell line and extracellular vesicles, and is not a cross-sectional study. Therefore we chose to analyze each biological sample in mass spectrometry technical triplicates.
Data exclusions	No data was excluded.
Replication	Two batches of extracellular vesicles were pooled and analysed. Technically, repeated MS injections were also used to control for technical variation. All experiments were replicated and reproducible from independent batches of cell material.
Randomization	Due to paired nature of cell line and extracellular vesicles, complete randomisation is not possible. Sample carryover was negligible, but EV samples were always injected before whole-cell samples in both proteome and ligandome analyses.
Blinding	Our samples involve paired analysis of cell lines and the extracellular vesicles secreted by these cell lines. Blinding is thus not possible.

Reporting for specific materials, systems and methods

We require information from authors about some types of materials, experimental systems and methods used in many studies. Here, indicate whether each material, system or method listed is relevant to your study. If you are not sure if a list item applies to your research, read the appropriate section before selecting a response.

Materials & experimental systems

n/a	Involved in the study
☐	☒ Antibodies
☐	☒ Eukaryotic cell lines
☒	☐ Palaeontology and archaeology
☒	☐ Animals and other organisms
☒	☐ Human research participants
☒	☐ Clinical data
☒	☐ Dual use research of concern

Methods

n/a	Involved in the study
☒	☐ ChIP-seq
☒	☐ Flow cytometry
☒	☐ MRI-based neuroimaging

Antibodies

Antibodies used	W6/32 was kindly provided by Dr. Stefan Stevanovic in Germany; CD81 (5A6, Santa Cruz Biotechnology, Santa Cruz CA)
Validation	W6/32 used in this work was supplied by Dr. Stefan Stevanovic in Germany. This is the source of antibodies featured in many of our publications (PMID: # 30784271; PMID: # 33087703) , as well as the publications of others in immunopeptidomics research. We have also verified that W6/32 correctly captures HLA class I proteins by mass spectrometry, and not BoLA proteins derived from bovine sources, as shown in this study (Supplementary Figure 5). CD81 - PMID: # 31303981; PMID: # 33935791; PMID: # 33850608;

Eukaryotic cell lines

Policy information about cell lines

Cell line source(s)	ATCC
Authentication	Cell lines were authenticated by ATCC at purchase and stocked upon purchase in multiple vials with passage number <7. Culture time of each stock vial was limited to 10 splittings, or 1.5 months for all experiments.
Mycoplasma contamination	Cell lines used were tested for mycoplasma contamination every 3 months by PCR, and verified negative.
Commonly misidentified lines (See ICLAC register)	Not applicable.
